# Food Additive Titanium Dioxide and Its Fate in Commercial Foods

**DOI:** 10.3390/nano9081175

**Published:** 2019-08-16

**Authors:** Ji-Soo Hwang, Jin Yu, Hyoung-Mi Kim, Jae-Min Oh, Soo-Jin Choi

**Affiliations:** 1 Division of Applied Food System, Major of Food Science & Technology, Seoul Women’s University, Seoul 01797, Korea; 2 Department of Chemistry and Medical Chemistry, College of Science and Technology, Yonsei University, Wonju 26493, Gangwondo, Korea; 3 Department of Energy and Materials Engineering, Dongguk University-Seoul, Seoul 04620, Korea

**Keywords:** titanium dioxide, nanostructure, size distribution, quantification, fate, commercial foods

## Abstract

Titanium dioxide (TiO_2_) is one of the most extensively utilized food additives (E171) in the food industry. Along with nanotechnology development, the concern about the presence of nanostructured particles in E171 TiO_2_ and commercial food products is growing. In the present study, the physicochemical properties of commercially available E171 TiO_2_ particles, including particle size distribution, were investigated, followed by their cytotoxicity and intestinal transport evaluation. The fate determination and quantification of E171 TiO_2_ in commercial foods were carried out based on the analytical procedure developed using simulated foods. The results demonstrated that TiO_2_ is a material mainly composed of particles larger than 100 nm, but present as an agglomerated or aggregated particle in commercial foods with amounts of less than 1% (wt/wt). Titanium dioxide particles generated reactive oxygen species and inhibited long-term colony formation, but the cytotoxicity was not related to particle size distribution or particle type (food- or general-grade). All TiO_2_ particles were mainly transported by microfold (M) cells, but also by intestinal tight junction. These findings will be useful for TiO_2_ application in the food industry and predicting its potential toxicity.

## 1. Introduction

Titanium dioxide (TiO_2_), naturally occurring oxide of titanium, is widely utilized as a food additive pigment in confectionary, such as chocolates, candies, bakeries, snacks, and chewing gums due to the fact of its brightening and whitening properties. In the European Union (EU), it is referred to as E171, with no maximum level specified [1,2,3]. The Joint Food and Agriculture Organization (FAO)/Word Health Organization (WHO) Expert Committee on Food Additives did not establish an acceptable daily intake (ADI) of TiO_2_, because its oral absorption amount is extremely low (maximum of 0.1%) [1,4,5]. In the United States, food additive TiO_2_ has been used since 1966, and the Food and Drug Administration (FDA) recommends its concentration, in general, below 1% by weight of the food [6,7]. Recent rapid developments in nanotechnology have led to the production of nano-sized TiO_2_, and it is probable that TiO_2_ nanoparticles (NPs) are imperceptibly used as food additive E171. However, the current specification for food additive TiO_2_ does not include characterization of particle size distribution. European Commission Recommendation 2011/696/EU defined a nanomaterial as a natural, incidental or manufactured material composed of more than 50% particles of 1–100 nm in the number–size distribution [8]. Recently, the *European Food Safety Authority (EFSA) Journal* reported that food additive E171 primarily consists of micro-sized TiO_2_ particles, with a nano-sized (<100 nm) particle less than 3.2% by mass or 10–39% by number–size distribution, obtained by scanning electron microscopy (SEM)/transmission electron microscopy (TEM) [1]. Yang et al. [9] also demonstrated that E171 TiO_2_ particles had 19–35% of nano-sized primary particles by number. In the current state, E171 TiO_2_ seems to not be considered as a nanomaterial according to the EU Recommendation. However, more extended study is required to assume that commercially available food additive E171 contains less than 50% of nano-sized TiO_2_ by number–size distribution. Moreover, information about the fate of TiO_2_ in commercial foods is necessary to be provided.

As concern about the use of NPs in food products grew, research on determination and quantification of food-grade TiO_2_ in commercial foods has recently increased [10,11,12]. However, the reliability of the methods used for TiO_2_ quantification has not been clearly demonstrated. Indeed, separation of TiO_2_ from complex food matrices and its quantification in the presence of food components are challenging, because diverse organic food matrices can disturb the accuracy and precision for the characterization and quantification of TiO_2_ [13,14]. In particular, NPs are generally known to be covered by environmental matrices, such as biological and food matrices, forming particle–matrix corona, which surely affects particle size distribution, surface characteristics, and fate [15,16,17,18]. Hence, it is necessary to establish an appropriate pretreatment method depending on food matrix type to dissolve organic matrices, which is critical for qualitative and quantitative analysis.

Studies have revealed that the oral absorption and bioavailability of TiO_2_, measured either as particles or as titanium, are extremely low [19,20,21,22]. Biokinetic behaviors of TiO_2_ were found to be independent of particle size [22], but highly affected by the presence of biomatrices [16]. The potential harmful effects of food-grade TiO_2_ on the intestinal epithelial barrier, intestinal disease, colorectal cancer, and development in offspring rodents have been recently reported [23,24,25,26]. Hence, further toxicity studies on food-grade TiO_2_ are urgently needed. Moreover, answers to the questions of whether food-grade TiO_2_ is more toxic than general-grade TiO_2_ and whether or not the former consists of smaller particle sizes than the latter are required.

In the present study, we characterized the physicochemical properties of food-grade E171 TiO_2_ commercially applied to food products. Pretreatment and analytical procedures for determination and quantification of TiO_2_ in food matrices were established using representative TiO_2_-containing simulated foods, and its identification was carried out in commercial foods. Finally, the cytotoxicity and intestinal transport mechanism of E171 TiO_2_ were evaluated and compared with those of general-grade bulk and nano TiO_2_ in human intestinal cells and in vitro 2D and 3D intestinal epithelium models, respectively.

## 2. Materials and Methods

### 2.1. Materials

Five food-grade E171 TiO_2_ products from four different manufacturers were provided as test materials and numbered as T1–T5. General-grade bulk and nano TiO_2_ were purchased from Sigma–Aldrich (St. Louis, MO, USA) and Alfa Aesar–Johnson Matthey Co. (Karlsruhe, Germany), respectively. Stock solutions (1 mg/mL) of particles were prepared by dispersion in distilled water (DW) for 30 min and sonication for 5 min, just prior to experiments. Commercial products containing TiO_2_ (chewing gum, candy, chocolate, jelly, sauces, and snacks) from international brands were purchased from markets in Seoul, Korea, in 2018.

### 2.2. Characterization

Primary particle size, morphology, and chemical characterization were determined by SEM (JSM-7100F, JEOL, Tokyo, Japan) equipped with energy-dispersive X-ray spectroscopy (EDS; Aztec, Oxford Instruments, Abingdon, UK). Zeta potentials and hydrodynamic radii of particles were measured with Zetasizer Nano System (Malvern Instruments, Worcestershire, UK). The X-ray diffraction (XRD) patterns were measured using an X-ray diffractometer (D2phaser, Bruker AXS Inc., Madison, WI, USA) with Ni-filtered CuKα radiation.

### 2.3. Size Fractionation

Fractionation of T4 particles depending on particle size was carried out using sucrose (Sigma–Aldrich) gradient solution. Four sucrose solutions (10, 20, 30, and 50%) were prepared and 10 mL of each sucrose solution was stacked in a conical tube (50 mL) from high to low concentration order. The T4 suspension (40 mg/mL) was prepared after stirring for 30 min and sonication for 5 min, and 10 mL of the suspension was carefully added on the top layer of sucrose gradient solution (10%). Fractionation of the particles was performed by centrifugation at 2000 rpm for 40 min. After centrifugation, the fractionated solution was divided into upper, middle, and lower parts, which were further characterized by SEM.

### 2.4. Preparation of TiO_2_-Containing Simulated Foods

Sugar powder containing TiO_2_ was prepared by mixing 4.9 g of grinded sugar (Samyang Co., Seoul, Korea) and 0.1 g of TiO_2_ in an agate mortar. Chewing gum containing TiO_2_ was prepared by adhering 0.01 g of TiO_2_ on the surface of 0.5 g of gum base, followed by surface coating with melted sugar. The contents of TiO_2_ in simulated foods were adjusted to 2% (wt/wt).

### 2.5. Pretreatment of Simulated Foods, Commercial Products, and Cell Samples for Characterization and Quantitative Analysis

Different pretreatment methods were applied depending on food type. One piece of simulated gum or commercial chewing gum in 15 mL of DW was stirred overnight. Thus, washed gum base in DW was collected and further washed with 5 mL of acetone (Samchun Pure Chemical Co., Ltd., Pyeongtaek, Gyeonggi-do, Korea) twice and 5 mL of ethanol (Samchun Pure Chemical Co., Ltd.), and then, all washed solutions were pooled. Finally, 1 mL of the pooled solution was pre-digested with nitric acid (Samchun Pure Chemical Co., Ltd.) and hydrogen peroxide (Samchun Pure Chemical Co., Ltd.).

The TiO_2_-containing simulated sugar powder, other commercial foods (0.2 g), such as candy, chocolate, jelly, sauces, and snacks, and the cell samples were directly subjected to pre-digestion with nitric acid and hydrogen peroxide. Nitric acid (10 mL) was added to each sample and heated to 180–200 °C in order to digest organic materials. Hydrogen peroxide (1 mL) was then added and heating was continued, and this procedure was repeated until the solution was colorless and clear. The pre-digested samples were resuspended in 1 mL of deionized and distilled water (DDW), and centrifuged at 10,000 rpm for 20 min. Then, the precipitate was washed twice with 2 mL of ethanol and re-suspended in 20 μL of ethanol for SEM-EDS analysis [27].

### 2.6. Quantitative Analysis

Sulfuric acid (10 mL, Samchun Pure Chemical Co., Ltd.) was added to the pre-digested samples and heated at 200 °C for 2 h in order to dissolve Ti from TiO_2_ particles and heated at 380 °C until the remaining solution was colorless and removed. The final digested and dissolved products were diluted to DDW (5 mL) and total Ti levels were analyzed by inductively coupled plasma-atomic emission spectroscopy (ICP-AES; JY2000 Ultrace, HORIBA Yvon Longjumeau, France).

### 2.7. Cell Culture

Human intestinal epithelial Caco-2 cells were supplied from the Korean Cell Line Bank (Seoul, Korea). The cells were cultured in minimum essential medium (MEM; Welgene, Gyeongsan, Korea), which contained 10% heat inactivated fetal bovine serum (FBS), penicillin (100 units/mL), and streptomycin (100 µg/mL, Welgene) in a 5% CO_2_ incubator at 37 °C.

### 2.8. Short-Term Cell Proliferation

The short-term effect of particles on cell proliferation was evaluated with water-soluble tetrazolium salt-1 (WST-1; Roche, Molecular Biochemicals, Mannheim, Germany). Cells (1 × 10^4^ cells/100 µL) were incubated with particles for 24 h. Then, WST-1 solution (10 µL) was added to each sample. After further incubation for 4 h, absorbance at 440 nm was measured using a microplate reader (SpectraMax^®^ M3, Molecular Devices). Cells incubated without particles were used as controls.

### 2.9. Cell Membrane Damage

The effect of particles on cell membrane integrity was monitored with a CytoTox 96 Non-Radioactive Cytotoxicity Assay. Cells (6 × 10^4^ cells/mL) were treated with particles (5, 50, 250, and 1000 µg/mL) for 24 h. Then, the cell culture medium was collected, centrifuged, and 50 µL of aliquots were used for analysis. Substrate solution (50 µL) was added to each aliquot, and further incubated for 30 min at room temperature. After addition of stop solution (50 µL) to the reacting solution, the absorbance at 490 nm was measured using a microplate reader (SpectraMax^®^ M3, Molecular Devices). Cells incubated without particles were used as controls.

### 2.10. Colony-Forming Ability

The long-term effect of particles on colony formation was evaluated by clonogenic assay. The cells (5 × 10^2^ cells/2 mL) were incubated with particles (5, 50, and 250 µg/mL) for 14 days. Then, the cells were washed with phosphate buffered saline (PBS; Dongin Biotech Co., Seoul, Republic of Korea), fixed with 90% methanol (Samchun Pure Chemical Co., Ltd.), and stained with 0.5% crystal violet solution (Sigma–Aldrich) for 1 h. After washing the cells with DDW and air-dying, colonies consisting of more than 50 cells were counted. Cells incubated without particles were used as controls.

### 2.11. Reactive oxygen species (ROS)

The intracellular ROS generated by particles was monitored with a peroxide-sensitive fluorescent probe, 2′,7′-dichlorofluorescein diacetate (H_2_DCFDA; Molecular Probes Inc., Eugene, OR, USA). Cells (1 × 10^4^ cells/100 µL) were treated with particles for 24 h, and then, 20 μM H_2_DCFDA were added and further incubated for 30 min at 37 °C in the dark. After washing with PBS, intracellular dichlorofluorescein fluorescence (DCF) was monitored using a fluorescence microplate reader (SpectraMax^®^ M3, Molecular Devices). Excitation and emission wavelengths were set at 485 and 535 nm, respectively.

### 2.12. Apoptosis Induction

The effect of particles on apoptosis induction was evaluated with annexin V-fluorescein isothiocyanate (FITC) and propidium iodide (PI), according to the manufacturer’s protocol (Invitrogen, Carlsbad, CA, USA). Cells (1 × 10^6^ cells) were exposed to particles (1 mg/mL). After incubation for 24 h, the cells were harvested with a scraper, washed with cold PBS, and then resuspended in binding buffer (cell density of 1 × 10^6^ cells/mL). Then, the cells were stained with annexin V-FITC (5 μL) and 1 μL of PI (100 μg/mL) working solution for 15 min at room temperature in the dark. After adding annexin-binding buffer (400 μL) to samples, the stained cells were finally measured with flow cytometry (Beckman Coulter, Brea, CA, USA) by counting 10,000 cells.

### 2.13. Intestinal Transportation

Human Burkitt’s lymphoma Raji B cells, supplied from the Korean Cell Line Bank, were cultured in Roswell Park Memorial Institute (RPMI) 1640 medium (Welgene), which contained FBS (10%), non-essential amino acids (1%), L-glutamine (1%), penicillin (100 units/mL), and streptomycin (100 μg/mL) in a 5% CO_2_ incubator at 37 °C. The follicle-associated epithelium (FAE) model, mimicking M cells, was prepared according to des Rieux et al. [28]. Briefly, Caco-2 cells (1 × 10^6^ cells/well) were cultured on upper inserts for 14 days. Raji B cells (1 × 10^6^ cells/well) in Dulbecco’s modified eagle’s medium (DMEM; Welgene) were then added to basolateral inserts, and co-cultured for 5 days (trans epithelial electrical resistance (TEER) of 150–200 Ω cm^2^). Finally, the apical medium of co-culture monolayers was replaced by medium containing particles (500 μg/mL) and further incubated for 6 h. The basolateral solutions were collected and used for quantitative analysis of transported TiO_2_ particles.

A Caco-2 monoculture model was used to evaluate particle transport by intestinal epithelial tight junction barrier. Caco-2 cells (4.5 × 10^5^ cells/well) were cultured on upper inserts for 21 days (TEER ≥ 300 Ω cm^2^), and apical medium of the monolayers was replaced by medium containing particles (500 μg/mL), and incubation was continued for 6 h. The basolateral solutions were collected to determine transported TiO_2_ concentration.

### 2.14. Statistical Analysis

Experimental data were presented as means ± standard deviations. One-way analysis of variance (ANOVA) followed by Tukey’s test in SAS Ver.9.4 (SAS Institute Inc., Cary, NC, USA) was carried out to determine statistical significance at *p*-values < 0.05.

## 3. Results

### 3.1. Characterization of Food Additive TiO_2_

**Figure 1:**
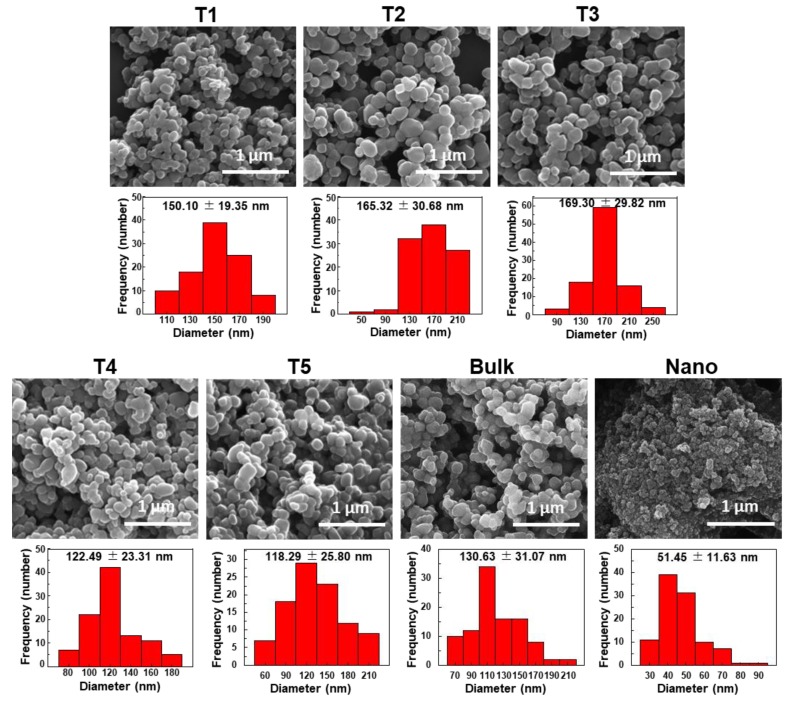
Scanning electron microscopy (SEM) images and size distributions of commercially available food additive TiO_2_ (T1–T5) and general-grade bulk and nano TiO_2_ (bulk and nano). Size distributions were obtained by randomly selecting 100 particles from the SEM images.

Crystal structure of commercially available food additive E171 TiO_2_ particles was determined by XRD patterns. All TiO_2_ particles were found to be anatase form without significant organic moiety (Figure S1). The SEM images showed irregular and randomly shaped particles, with average primary particle sizes of 118.29 ± 25.80–169.30 ± 29.82 nm and particle size distributions of 1–22% of smaller than 100 nm (Figure 1 and Table 1). Statistical differences in average primary particle size among five food additive TiO_2_ particles were not found (*p* > 0.05). Meanwhile, general-grade nano TiO_2_ had average particle sizes of 51.45 ± 11.63 consisting of 100% smaller than 100 nm. Whereas, general-grade bulk TiO_2_ had statistically similar particle sizes compared to food additive TiO_2_ particles.

Dynamic light scattering (DLS) results demonstrated that the Z-average sizes of TiO_2_ particles in DW were 285.30 ± 6.68–345.27 ± 4.45 nm, and 100% of the hydrodynamic diameters of all particles were larger than 100 nm (Table 2). Thus, the hydrodynamic radii of TiO_2_ particles in aqueous solution were much larger than the primary particle sizes measured by SEM (Figure 1). Zeta potentials of all food additive TiO_2_ particles were negative, showing −36.70 ± 2.01 to −42.17 ± 3.61 mV (Table 2). On the other hand, size distribution of general-grade bulk TiO_2_, measured by DLS, was 45.47% of 100–200 nm and 54.53% of larger than 200 nm, without particle size smaller than 100 nm, whereas, general-grade nano TiO_2_ was composed of 38.27% of smaller than 100 nm, 61.60% of 100–200 nm, and minimum level (0.13%) of larger than 200 nm. Considering average particle sizes and size distributions obtained by SEM and DLS analysis, T3 and T4 were used as representative largest and smallest particles, respectively, for further study.

### 3.2. Characterization and Quantitative Analysis of TiO_2_ in Simulated Foods

**Figure 2:**
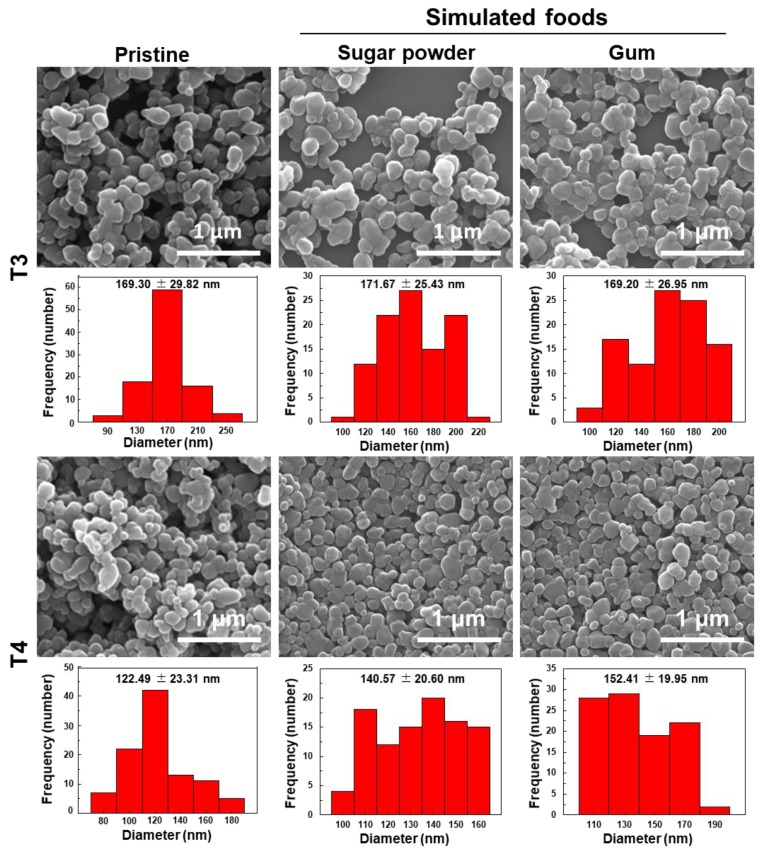
Scanning electron microscopy (SEM) images and size distributions of food additive TiO_2_ (T3 and T4) recovered from simulated foods. Size distributions were obtained by randomly selecting 100 particles from SEM images.

In order to check the reliability of analytical procedure for detection of TiO_2_ in commercial food products, representative TiO_2_-containing simulated foods, such as sugar powder and chewing gum, were prepared based on the major utilization of TiO_2_ in foods. The simulated foods were prepared by mixing TiO_2_ with grinded sugar powder or by attaching TiO_2_ on the surface of gum base followed by sugar coating. After acid (nitric acid and hydrogen peroxide) treatment for digestion of organic matrices, SEM images showed that TiO_2_ particles (T3 and T4) recovered from simulated foods had almost the same morphology and particle size distribution compared to pristine ones (Figure 2). The DLS results demonstrated no statistical differences in hydrodynamic radii among pristine TiO_2_ and TiO_2_ recovered from simulated foods (Table 3). On the other hand, zeta potential values of TiO_2_ recovered from simulated foods changed to be fewer negative charges compared to pristine TiO_2_ (Table 3).

**Figure 3:**
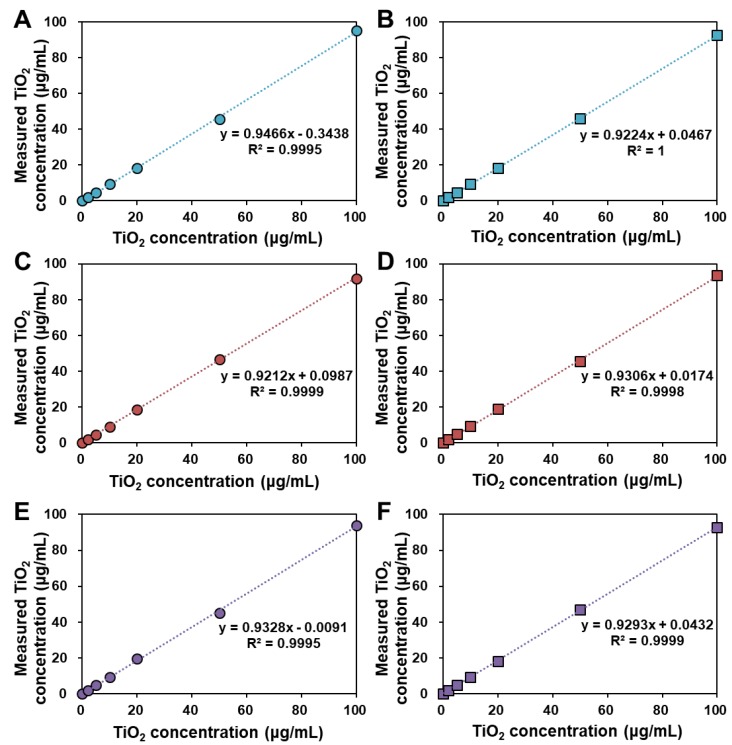
Calibration curves for determination of (**A**) pristine T3 and (**B**) pristine T4, (**C**) T3 and (**D**) T4 in simulated sugar powder, and (**E**) T3 and (**F**) T4 in simulated gum by inductively coupled plasma-atomic emission spectroscopy (ICP-AES) analysis.

Standard calibration curves of TiO_2_ (T3 and T4) in the simulated foods were obtained after acid digestion of food matrices, dissolution of TiO_2_ in sulfuric acid, followed by Ti quantification by ICP-AES. In this step, sulfuric acid was used to dissolve Ti from TiO_2_ [29,30]. Figure 3 showed that there were no significant differences in Ti quantification between pristine TiO_2_ and sugar powder- or gum base-spiked TiO_2_, with linear correlation coefficients. The recoveries (%) of TiO_2_ were more than 90% in all cases, regardless of particle size distribution (T3 and T4) and food matrix type (Table 4). The limits of detection (LOD) and limits of quantification (LOQ) for pristine TiO_2_ as well as TiO_2_ in simulated foods were sufficiently low to detect the particles in food matrices.

### 3.3. Characterization and Quantitative Analysis of TiO_2_ in Commercial Foods

**Figure 4:**
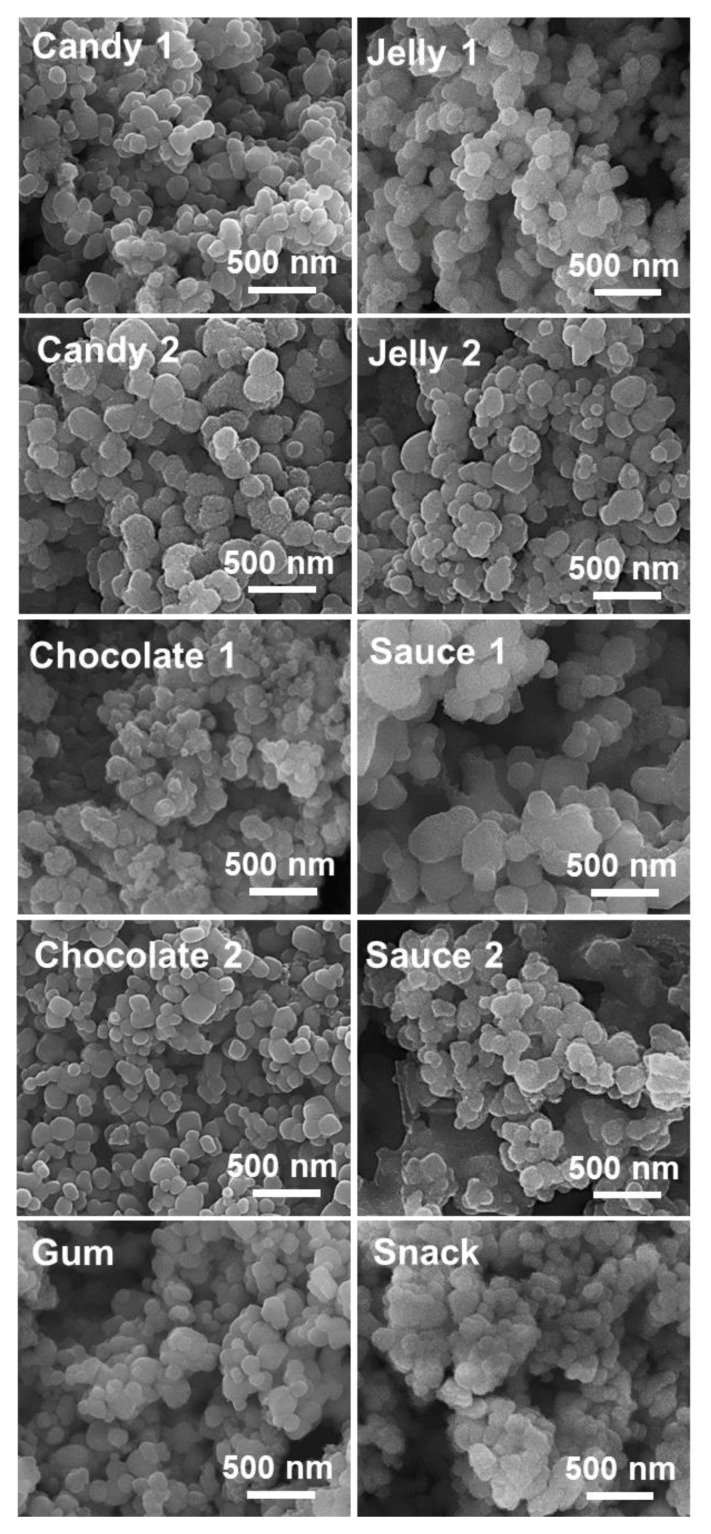
Scanning electron microscopy (SEM) images of TiO_2_ particles recovered from commercial foods.

Food additive TiO_2_ particles were characterized and identified from various commercial products, such as candy, chocolate, chewing gum, jelly, sauces, and snacks, based on the method established with the simulated foods. The SEM images revealed that TiO_2_ particles recovered from commercial foods by nitric acid/hydrogen peroxide treatment had similar shape and particle size distribution (Figure 4) without significant decomposition compared to pristine TiO_2_ (Figure 1). The DLS results showed that the hydrodynamic radii of TiO_2_ particles recovered from commercial products were almost similar, ranging from 242.53 ± 45.60–461.47 ± 6.76 nm (Table 5), compared to those of pristine (Table 2). Zeta potential changes to less negative charges were also observed (Table 5), as did in simulated foods (Table 3). Element analysis using EDS confirmed the presence of Ti in the particles recovered from commercial foods (Figure S2).

On the other hand, quantitative analysis revealed that TiO_2_ contents ranged from 1.09–9.87 mg/g, representing 0.11–0.99% (wt/wt) (Table 5).

### 3.4. Size Fractionation of TiO_2_

**Figure 5:**
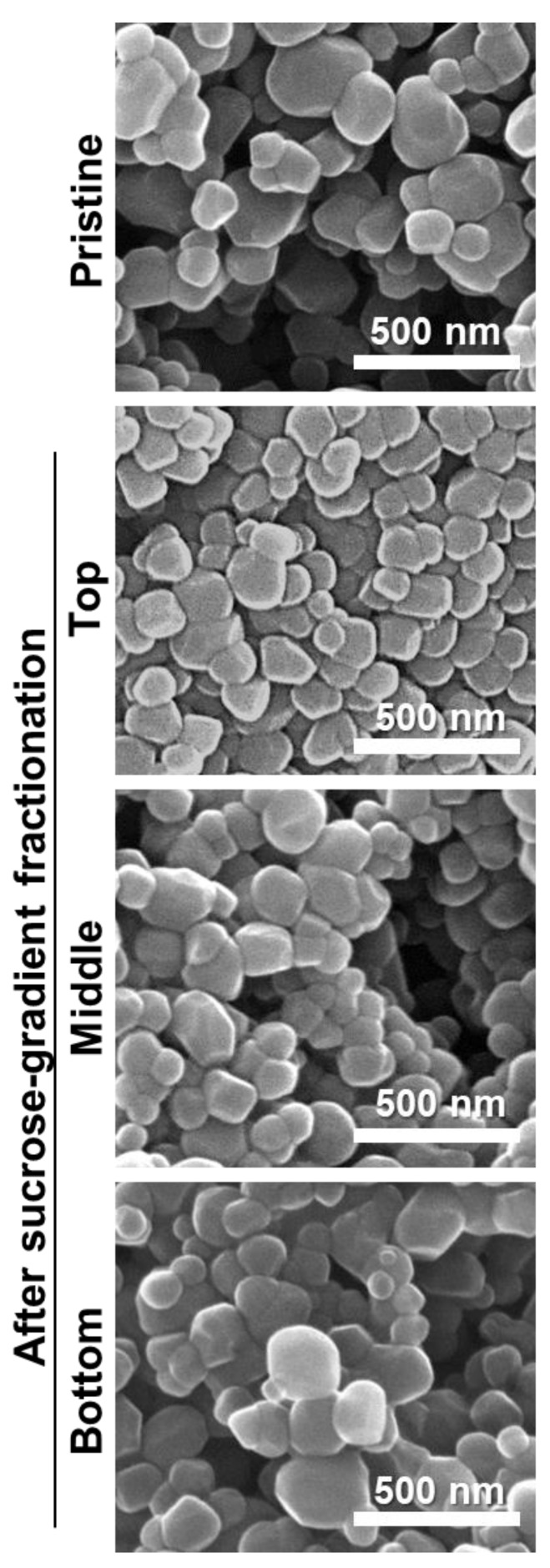
Scanning electron microscopy (SEM) images of TiO_2_ (T4) in the top, middle, and bottom layers fractionated by a sucrose gradient method.

Size fractionation was performed with the smallest particle T4 using sucrose gradient solution in order to obtain smaller particle size. Figure 5 demonstrated that the top layer of T4 after sucrose gradient fractionation had smaller size with narrower size distribution (96.94 ± 17.86 nm, Table S1) than pristine T4 (122.49 ± 23.31 nm), and the particle size increased in the bottom layer. The homogeneity of size distribution was checked by kurtosis values from normal distribution fitting (Table S1). Kurtosis value for the top layer was 1.11, more positive than the value (1.07) for pristine T4, indicating narrower size distribution of the former than the latter. Thus, the top layer of T4 after size fractionation was used for further cytotoxicity study.

### 3.5. Inhibition of Cell Proliferation, Membrane Damage, and Colony Formation

**Figure 6:**
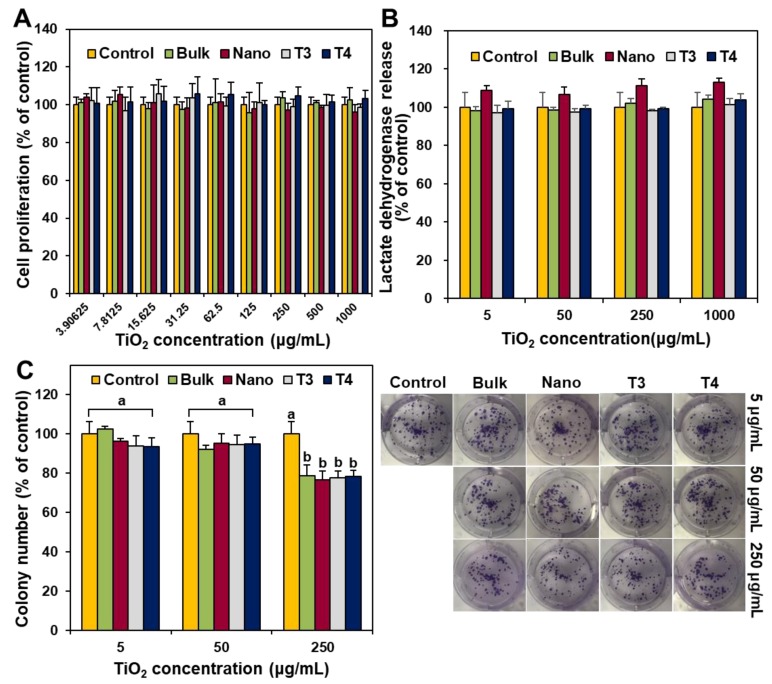
Short-term effect of TiO_2_ particles on (**A**) cell proliferation and (**B**) membrane damage of human intestinal Caco-2 cells after 24 h. (**C**) Long-term effect of TiO_2_ particles on colony-forming ability of Caco-2 cells after 14 days. Different lowercase letters (a,b) indicate significant differences among control (cells without particle) and different TiO_2_ particles (food-grade T3, T4, general-grade bulk and nano) (*p* < 0.05).

The effects of food additive TiO_2_ with different size distributions (T3 and T4) on short-term cell proliferation and membrane damage were evaluated in human intestinal Caco-2 cells after 24 h exposure with WST-1 and lactate dehydrogenase (LDH) leakage assays, respectively. General-grade bulk and nano TiO_2_ were also used for comparative study. The results demonstrated that all food-grade and general-grade TiO_2_ particles did not affect cell proliferation or membrane integrity after 24 h (Figure 6A,B).

The long-term effect of food additive TiO_2_ on colony formation was also investigated with clonogenic assay and compared with general-grade bulk and nano TiO_2_. Figure 6C showed that all TiO_2_ particles slightly inhibited colony-forming ability at the highest concentration tested (250 μg/mL) after exposure for 14 days, regardless of particle size distribution or type.

**Figure 7:**
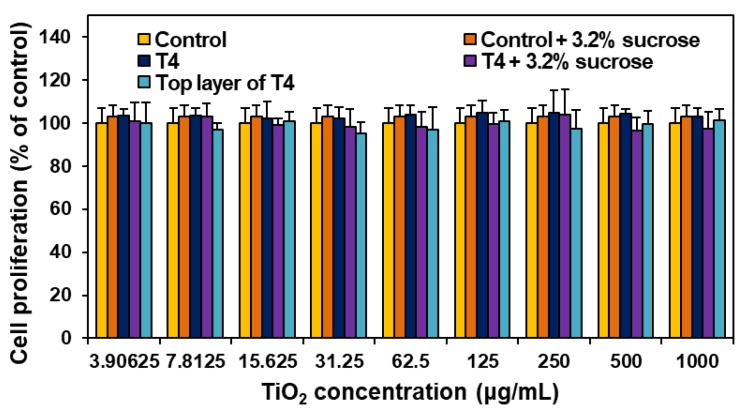
The effect of the smallest particle fraction of TiO_2_ (T4), the top layer after size fractionation, on cell proliferation of Caco-2 cells after 24 h. No significant differences among controls (cells without particles), T4, and top layer of T4 were found (*p* > 0.05).

When the effect of the smallest particle fraction of T4 (Figure 5), the top layer after size fractionation using sucrose gradient solution, on short-term cell proliferation, was evaluated with the WST-1 assay, no significant differences in cytotoxicity were found between pristine T4 and size-fractioned top layer of T4 (Figure 7). Cell culture medium or T4 containing 3.2% sucrose (the remaining sucrose concentration after size fractionation) was also used as controls to confirm no effect of the sucrose on cytotoxicity.

### 3.6. ROS and Apoptosis Induction

**Figure 8:**
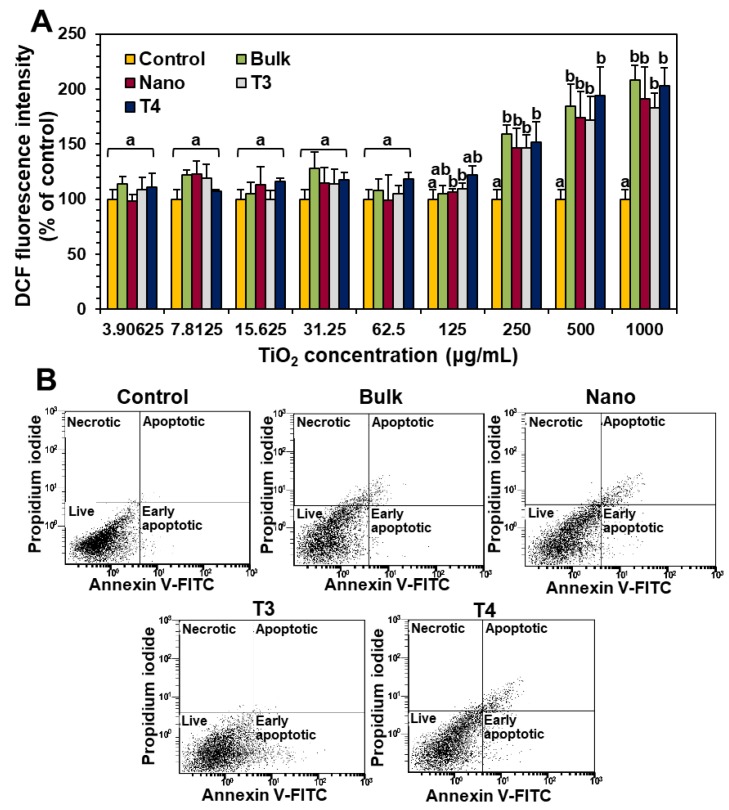
The effect of TiO_2_ particles on (**A**) intracellular reactive oxygen species (ROS) generation and (**B**) apoptosis induction in Caco-2 cells after 24 h. Different lowercase letters (a, b) indicate significant differences among control (cells without particles) and different TiO_2_ particles (food-grade T3, T4, general-grade bulk and nano) (*p* < 0.05).

The potential of food additive TiO_2_ (T3 and T4) on oxidative stress induction was evaluated by measuring intracellular ROS. Apoptosis induction was also identified with annexin V-FITC, which binds to the phosphatidylserine exposed at cell surface, that represents an early marker of apoptosis. PI, a dye which is not taken up by the viable cells, was used as a dead cell marker. The results showed that all food-grade TiO_2_ and general-grade bulk and nano TiO_2_ significantly induced ROS at above 125 µg/mL after 24 h exposure (Figure 8A). No significant differences in ROS generation were found among different types of particles (*p* > 0.05).

On the other hand, all TiO_2_ particles slightly caused apoptosis or necrosis at the highest concentration tested, 1000 µg/mL after exposure for 24 h (Figure 8B), showing total apoptotic or necrotic cells of ~7.9, 11.9, 6.8, and 12.5% for general-grade bulk, general-grade nano, T3, and T4 TiO_2_ particles, respectively.

### 3.7. Intestinal Transport Mechanism

**Figure 9:**
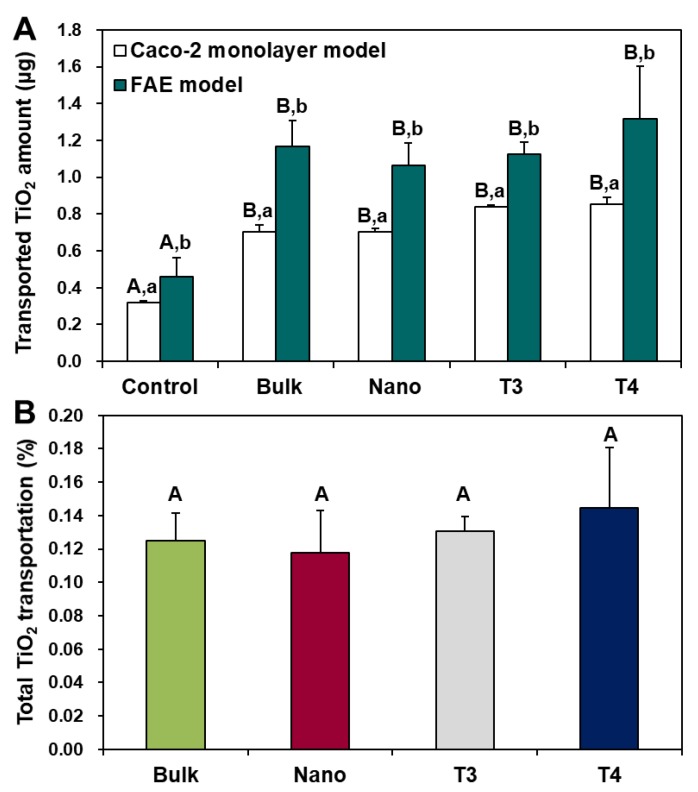
Intestinal transport mechanism of TiO_2_ particles using (**A**) in vitro models of Caco-2 monolayer and human follicle-associated epithelium (FAE). (**B**) Total combined intestinal transport amount of TiO_2_ particles through both Caco-2 monolayer and FAE models. Different uppercase letters (A, B) indicate significant differences among control (cells without particles) and different TiO_2_ particles (food-grade T3, T4, general-grade bulk and nano) (*p* < 0.05). Different lowercase letters (a, b) indicate significant differences between Caco-2 monolayer and FAE models (*p* < 0.05).

Particles must be transported across the intestinal epithelial barrier to be absorbed. The intestinal transport mechanism of food additive TiO_2_ (T3 and T4) and general-grade bulk and nano TiO_2_ was investigated by two different culture models: a 2D model, the Caco-2 monolayer, and a 3D one, the FAE. The former and the latter represent microfold (M) cells found in FAE overlying Peyer’s patches and intestinal tight junction, respectively. The results demonstrated that all particles were primarily transported by M cells, but significantly increased transports through Caco-2 monolayer were also found (Figure 9A). The total transport amounts of all TiO_2_ particles, combined transportation by both M cells and the Caco-2 monolayer, was extremely low, showing 0.12–0.14% (Figure 9B). The effects of particle size or particle type (food- or general-grade) on intestinal transport amount and mechanism were not found (*p* > 0.05).

## 4. Discussion

Titanium dioxide is a widely utilized food additive in the food industry. Hence, the characterization of food additive TiO_2_ and its determination in commercial foods are of importance, which can answer the fundamental question as to whether food additive TiO_2_ particles consist of nano-sized materials or not. In the present study, commercially available food additive E171 TiO_2_ particles were found to be composed of particle sizes of mainly larger than 100 nm, showing 1–22% smaller than 100 nm in number–size distribution, based on SEM images (Figure 1 and Table 1). On the other hand, DLS results demonstrated that the hydrodynamic diameters of all food additive TiO_2_ particles were larger than 100 nm and that the hydrodynamic radii increased in DW (Table 2) compared to primary particle sizes obtained by SEM analysis (Figure 1). This result suggests that TiO_2_ particles were present as agglomerated or aggregated forms under aqueous condition. Taken together, TiO_2_ particles are not considered as nanomaterials according to European Commission Recommendation 2011/696/EU [8].

For accurate and reliable TiO_2_ determination and quantification in commercial foods, we first established the analytical method using representative TiO_2_-containing simulated foods, such as sugar powder and gum. Nitric acid and hydrogen peroxide treatment was performed to digest organic food matrices [31,32], which permits physicochemical characterization of intact TiO_2_ particles after recovery. The results demonstrated almost similar shape and particle size of TiO_2_ recovered from the simulated foods compared to pristine TiO_2_ (Figure 2 and Table 3). This indicates that nitric acid/hydrogen peroxide digestion did not significantly alter the morphology and particle size distribution of TiO_2_. It is known that TiO_2_ is resistant to acids, except concentrated sulfuric acid [29,30]. Zeta potential changes of TiO_2_ to fewer negative values after recovery from the simulated foods are strongly likely to be related to acid treatment. It was reported that nitric acid-treated TiO_2_ particles are positively charged by adsorbing nitric acid on the surface [33,34]. Although nitric acid was evaporated by heating, and then, TiO_2_ particles were dispersed in DW or ethanol for physicochemical characterization, it is probable that a small portion of nitric acid remained, affecting the zeta potential values. Meanwhile, sulfuric acid treatment was used for Ti dissolution from TiO_2_, which contributes to Ti quantification by ICP-AES [29,30,35,36]. Figure 3 and Table 4 clearly showed that nitric acid/hydrogen peroxide/sulfuric acid pretreatment can be used as an accurate and precise method for TiO_2_ quantification.

When the above method was applied to recover TiO_2_ particles from diverse commercial foods, almost similar morphology and particle size compared to pristine TiO_2_ were observed (Figure 4), although slightly increased hydrodynamic radii of TiO_2_ were found in some cases (Table 5). These results suggest that TiO_2_ particles were present as intact particle forms but could be more agglomerated or aggregated to some extent in food matrices. It is worth noting that the presence of Ti in the particles recovered from commercial products was confirmed by EDS analysis (Figure S2). Based on the results obtained by pristine TiO_2_, TiO_2_-containing simulated foods, and TiO_2_-containing commercial foods, TiO_2_ is a material primarily composed of larger than 100 nm and present as an agglomerated or aggregated particle in aqueous solution or food matrices. On the other hand, quantitative analysis revealed that less than 1% (wt/wt) TiO_2_ was detected in all commercial food products, which is well correlated to the FDA recommendation [7].

TiO_2_ particles (T3 and T4) did not cause short-term inhibition of cell proliferation (Figure 6A) or membrane damage (Figure 6B), but slightly induced apoptosis (Figure 8B) after 24 h exposure, as general-grade bulk or nano TiO_2_ did. When further cytotoxicity was evaluated with smaller and narrower size distribution of T4 after sucrose gradient fractionation (Figure 5 and Table S1), no effect on short-term cell proliferation was also found (Figure 7). However, long-term colony formation was inhibited by high concentration (250 μg/mL) of all TiO_2_ particles after exposure for 14 days (Figure 6C). Although long-term exposure time and high concentration were used in the point of view of digestion physiology, particle accumulation at target specific organs for prolonged time could not be completely excluded. Nevertheless, it is worth noting that the concentrations exhibiting cytotoxicity are too high to be taken up by food-additive TiO_2_ in commercial products, considering its extremely low oral absorption. On the other hand, TiO_2_ particles generated ROS at more than 125 μg/mL after 24 h (Figure 8A), regardless of particle size distribution (T3 or T4) or particle type (food- or general-grade). It is likely that the size is not a critical factor affecting the cytotoxicity of TiO_2_. Other reports demonstrated that TiO_2_ particles generated ROS but did not affect cell viability at similar concentrations used in this study [37,38,39]. High ROS generation but relatively low apoptosis induction by TiO_2_ particles were also reported [40,41,42], which is consistent with our results. Indeed, ROS generation and oxidative stress play roles in apoptosis induction [43,44], but more exposure time or high concentration seems to be needed to examine apoptosis. The TiO_2_ particles were reported to cause oxidative stress [9], which may lead to DNA damage, inflammation, and possibly cancer [45,46,47]. Hence, the cytotoxicity potential of TiO_2_ is likely to be associated with its chemical characteristics, not with particle size. Further study is required to elucidate the toxicity mechanism and chronic toxicity potential of food-additive TiO_2_ particles.

On the other hand, the intestinal transport mechanism of TiO_2_ was determined to be by both M cells and Caco-2 monolayer, and its primary transportation route was by M cells, showing extremely low transport amount (total 0.12–0.14%) (Figure 9). The result obtained in in vitro 2D and 3D culture models was highly consistent with in vivo absorption after oral administration of food- or general-grade TiO_2_ particles to rats [16,22]. It is important to note that particle size distribution or particle type did not significantly affect the intestinal transport amount and mechanism of TiO_2_ particles.

## 5. Conclusions

In this study, commercially available food additive TiO_2_ particles were characterized and identified in commercial foods. The results obtained demonstrated that TiO_2_ was a material mainly consisting of larger than 100 nm, and its fate in commercial foods was an agglomerated or aggregated particle, without significant decomposition in food matrices. Hence, E171 TiO_2_ is not a nanostructured material. Less than 1% TiO_2_ were quantitatively detected in all commercial food products. The cytotoxicity of TiO_2_ was mainly associated with ROS generation and long-term inhibition of colony-forming ability only at high concentration, but particle size distribution or particle type (food- or general-grade) did not affect the cytotoxicity or intestinal transport amount and mechanism. These findings will be useful for TiO_2_ application in the food industry and predicting its potential toxicity.

## Figures and Tables

**Table 1:** Primary particle sizes and size distributions of food additive TiO_2_ (T1–T5) and general-grade bulk and nano TiO_2_ (bulk and nano) measured by SEM.

Sample	Average Size (nm)	Distribution (No. %)		
		<100 nm	100–200 nm	>200 nm
T1	150.10 ± 19.35 ^b^	1	96	3
T2	165.32 ± 30.68 ^b^	3	80	17
T3	169.30 ± 29.82 ^b^	1	76	23
T4	122.49 ± 23.31 ^b^	12	88	ND
T5	118.29 ± 25.80 ^ab^	22	74	4
Bulk	130.63 ± 31.07 ^b^	14	84	2
Nano	51.45 ± 11.63 ^a^	100	ND	ND

Different lowercase letters (a, b) indicate significant differences among different TiO_2_ particles (*p* < 0.05). No., number; ND, not detectable.

**Table 2:** Size distributions, hydrodynamic radii, and zeta potentials of food additive TiO_2_ (T1–T5) and general-grade bulk and nano TiO_2_ (bulk and nano).

Sample	Distribution (No. %)			Z-Average Size (nm)	Zeta Potential (mV)
	<100 nm	100–200 nm	>200 nm		
T1	ND	44.73 ± 7.05	55.27 ± 7.05	285.30 ± 6.68 ^b^	−40.50 ± 3.06 ^a^
T2	ND	38.97 ± 1.32	61.03 ± 1.32	299.93 ± 1.79 ^b^	−40.73 ± 2.46 ^a^
T3	ND	10.43 ± 1.40	89.57 ± 1.40	345.27 ± 4.45 ^c^	−42.17 ± 1.63 ^a^
T4	ND	49.97 ± 4.87	50.03 ± 4.87	291.60 ± 3.76 ^b^	−36.70 ± 2.01 ^a^
T5	ND	12.53 ± 2.61	87.47 ± 2.61	340.00 ± 3.94 ^c^	−42.17 ± 3.61 ^a^
Bulk	ND	45.47 ± 1.70	54.53 ± 1.70	293.10 ± 0.52 ^b^	−37.90 ± 3.38 ^a^
Nano	38.27 ± 18.28	61.60 ± 18.10	0.13 ± 0.23	153.43 ± 11.72 ^a^	13.77 ± 0.74 ^b^

Different lowercase letters (a, b, c) indicate significant differences among different particles (*p* < 0.05). No., number; ND, not detectable.

**Table 3:** Size distributions, hydrodynamic radii, and zeta potentials of food additive TiO_2_ (T3 and T4) recovered from simulated foods.

Sample		Distribution (No. %)			Z-Average Size (nm)	Zeta Potential (mV)
		<100 nm	100–200 nm	>200 nm		
**T3**	Pristine	ND	10.43 ± 1.40	89.57 ± 1.40	345.27 ± 4.45 ^a^	−42.17 ± 1.63 ^a^
	Sugar powder	ND	37.03 ± 13.22	62.97 ± 13.22	327.30 ± 14.90 ^a^	−21.77 ± 6.61 ^b^
	Gum	ND	24.97 ± 3.39	75.03 ± 3.39	332.30 ± 5.20 ^a^	−17.37 ± 1.99 ^b^
**T4**	Pristine	ND	49.97 ± 4.87	50.03 ± 4.87	291.60 ± 3.76 ^a^	−36.70 ± 2.04 ^a^
	Sugar powder	0.23 ± 0.40	36.83 ± 26.04	62.93 ± 26.28	293.27 ± 16.69 ^a^	−37.67 ± 3.48 ^a^
	Gum	ND	40.07 ± 17.56	59.93 ± 17.56	315.40 ± 2.46 ^a^	−16.57 ± 1.12 ^b^

Different lowercase letters (a, b) indicate significant differences between pristine TiO_2_ and TiO_2_ recovered from simulated foods (*p* < 0.05). No., number; ND, not detectable.

**Table 4:** Analytical recovery, limit of detection (LOD), and limit of quantification (LOQ) of quantitative analytical procedure for TiO_2_.

Sample		Parameters	TiO_2_ Concentration (μg/mL)					
			2	5	10	20	50	100
Pristine	T3	Recovery (%)	93.42 ± 2.78	93.86 ± 7.43	92.14 ± 3.53	91.34 ± 1.52	90.88 ± 5.72	95.12 ± 1.52
		LOD (μg/mL)	0.05					
		LOQ (μg/mL)	0.15					
	T4	Recovery (%)	102.08 ± 1.67	93.31 ± 4.60	95.09 ± 4.66	91.18 ± 0.57	91.88 ± 6.39	92.43 ± 2.54
		LOD (μg/mL)	0.23					
		LOQ (μg/mL)	0.70					
Simulated sugar powder	T3	Recovery (%)	96.81 ± 3.48	93.19 ± 10.98	90.85 ± 8.49	92.72 ± 3.49	93.65 ± 4.24	91.91 ± 2.80
		LOD (μg/mL)	0.34					
		LOQ (μg/mL)	1.04					
	T4	Recovery (%)	99.15 ± 2.64	97.96 ± 3.59	92.12 ± 3.96	95.01 ± 2.57	91.10 ± 7.21	93.50 ± 3.67
		LOD (μg/mL)	0.18					
		LOQ (μg/mL)	0.55					
Simulated gum	T3	Recovery (%)	93.96 ± 4.89	93.96 ± 3.50	93.41 ± 3.01	97.72 ± 2.34	90.03 ± 5.48	93.90 ± 0.64
		LOD (μg/mL)	0.03					
		LOQ (μg/mL)	0.09					
	T4	Recovery (%)	97.58 ± 3.64	96.30 ± 3.98	93.74 ± 2.35	90.54 ± 1.16	94.04 ± 2.82	92.81 ± 1.88
		LOD (μg/mL)	0.21					
		LOQ (μg/mL)	0.62					

**Table 5:** Size distributions, hydrodynamic radii, zeta potentials, and quantitative results of TiO_2_ recovered from commercial foods.

Commercial Products	Distribution (No. %)			Z-Average Size (nm)	Zeta Potential (mV)	TiO_2_ Contents (mg/g)
	<100 nm	100–200 nm	>200 nm			
Candy 1	ND	ND	100.00 ± 0.00	417.80 ± 25.06	−35.37 ± 2.03	1.09 ± 0.03
Candy 2	3.83 ± 6.64	39.67 ± 25.52	56.50 ± 31.92	306.83 ± 59.91	−13.13 ± 0.45	9.87 ± 0.21
Chocolate 1	2.10 ± 3.64	25.93 ± 37.80	71.97 ± 35.75	242.53 ± 45.60	−12.03 ± 2.83	8.63 ± 0.04
Chocolate 2	ND	27.03 ± 13.11	72.97 ± 13.11	264.97 ± 14.55	−31.87 ± 1.50	6.32 ± 0.63
Gum	11.73 ± 16.82	47.50 ± 14.81	40.77 ± 22.17	344.90 ± 4.78	−14.50 ± 0.62	3.53 ± 0.12
Jelly 1	0.90 ± 1.56	35.47 ± 27.51	63.63 ± 28.93	363.17 ± 11.29	−37.23 ± 0.72	1.30 ± 0.13
Jelly 2	ND	17.43 ± 23.88	82.57 ± 23.88	298.83 ± 82.98	−32.57 ± 0.76	2.33 ± 0.37
Sauce 1	ND	63.40 ± 34.26	36.60 ± 34.26	316.07 ± 35.30	−25.20 ± 1.15	3.70 ± 0.41
Sauce 2	ND	11.33 ± 17.76	88.67 ± 17.76	461.47 ± 6.76	−36.80 ± 0.78	0.94 ± 0.04
Snack	ND	2.87 ± 4.13	97.13 ± 4.13	356.63 ± 8.45	−25.93 ± 0.47	1.27 ± 0.08

No., number; ND, not detectable.

## Supplementary Materials

The following are available online at https://www.mdpi.com/2079-4991/9/8/1175/s1. Figure S1: X-ray diffraction (XRD) patterns of commercially available food additive TiO_2_ (T1–T5) particles; Figure S2: Energy-dispersive X-ray spectroscopy (EDS) spectra of particles recovered from commercial foods; Table S1: Normal distribution fitting result for T4 before and after size fractionation using sucrose gradient solution.
