# Supplementary material for: Food Additive Titanium Dioxide and Its Fate in Commercial Foods
Source: Nanomaterials (Basel). 2019 Aug 16;9(8):1175. doi: 10.3390/nano9081175 (PMC6724087; doi:10.3390/nano9081175)
Supplement: Supplementary file 1 [file nanomaterials-09-01175-s001.pdf]

Article

Food Additive Titanium Dioxide and Its Fate in Commercial Foods

Ji-Soo Hwang [1], Jin Yu [1], Hyoung-Mi Kim [2], Jae-Min Oh [3] and Soo-Jin Choi [1,*]

[1] Division of Applied Food System, Major of Food Science & Technology, Seoul Women's University, Seoul 01797, Korea

[2] Department of Chemistry and Medical Chemistry, College of Science and Technology, Yonsei University, Wonju 26493, Gangwondo, Korea

[3] Department of Energy and Materials Engineering, Dongguk University-Seoul, Seoul 04620, Korea

* Correspondence: sjchoi@swu.ac.kr; Tel.: +82-2-970-5634; Fax: +82-2-970-5977

Received: 30 July 2019; Accepted: 13 August 2019; Published: date

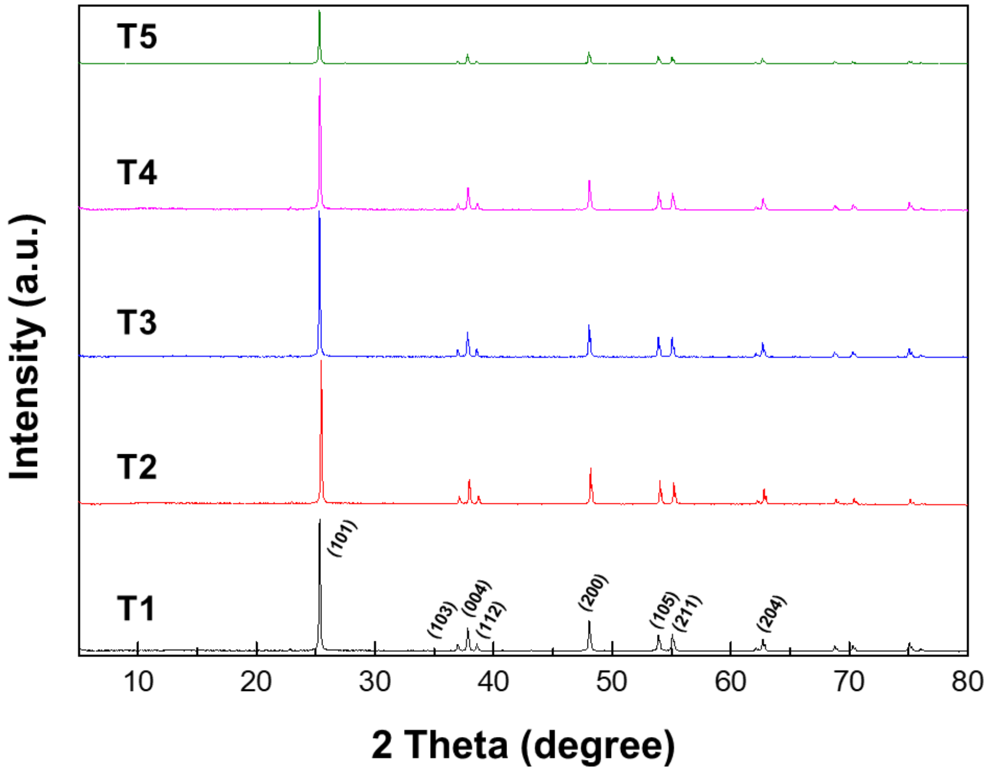

Figure S1. X-ray diffraction (XRD) patterns of commercially available food additive TiO_2 (T1–T5) particles.

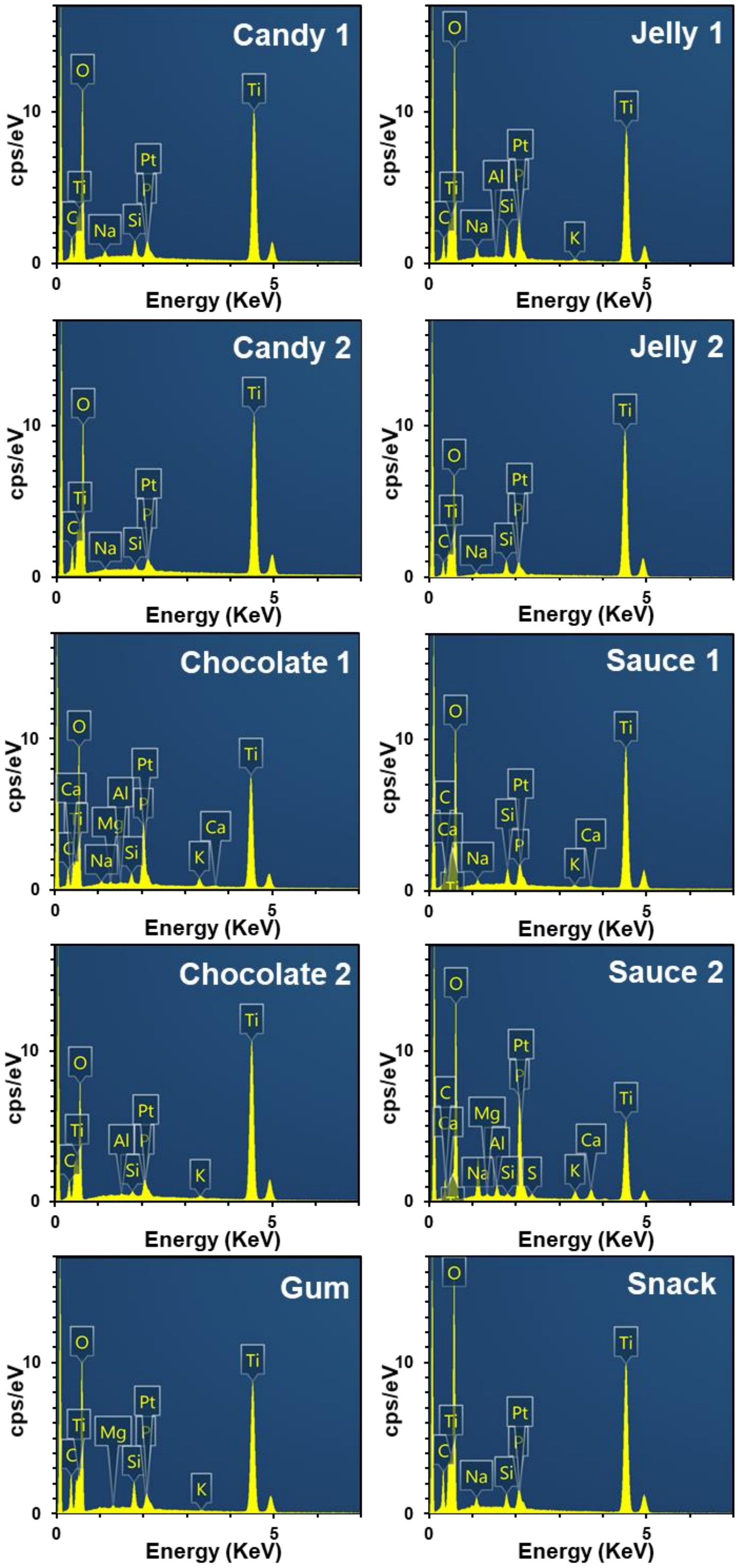

Figure S2. Energy dispersive X-ray spectroscopy (EDS) spectra of particles recovered from commercial foods.

Table S1. Normal distribution fitting result for T4 before and after size fractionation using sucrose gradient solution.

**Sample**		**Average size (nm)***	**Kurtosis**
Pristine		122.49 ± 23.31 ab	1.07
After size fractionation	Top layer	96.94 ± 17.86 a	1.11
	Middle layer	140.42 ± 26.38 ab	-1.41
	Bottom layer	179.51 ± 36.40 b	-2.41

*Average particle sizes were obtained from scanning electron microscopy (SEM) images. Different lower-case letters (a, b) indicate significant differences among pristine TiO_2 (T4) and fractionated TiO_2 particles ($P < 0.05$).
